# Epidermal Neural Crest Stem Cell Conditioned Medium Enhances Spinal Cord Injury Recovery via PI3K/AKT-Mediated Neuronal Apoptosis Suppression

**DOI:** 10.1007/s11064-024-04207-8

**Published:** 2024-07-18

**Authors:** Ziqian Ma, Tao Liu, Liang Liu, Yilun Pei, Tianyi Wang, Zhijie Wang, Yun Guan, Xinwei Zhang, Yan Zhang, Xueming Chen

**Affiliations:** 1 Department of Orthopedics Surgery, Beijing Luhe Hospital, Capital Medical University, Beijing, China; 2 Department of Orthopedics, 981st Hospital of the Chinese People’s Liberation Army Joint Logistics Support Force, Chengde, 067000 Hebei Province P.R. China; 3 Department of Pediatric Internal Medicine, Affiliated Hospital of Chengde Medical University, Chengde, China; 4 Department of Anesthesiology and Critical Care Medicine, School of Medicine, Johns Hopkins University, Baltimore, MD USA; 5 Department of Neurological Surgery, School of Medicine, Johns Hopkins University, Baltimore, MD USA; 6 Department of Orthopedics, Beijing Chaoyang Hospital, Capital Medical University, 8 Workers Stadium South Road, Chaoyang District, Beijing, China

**Keywords:** Spinal cord injury, Epidermal neural crest stem cells, Condition medium, Neuronal apoptosis, SHSY-5Y cell line, PI3K/AKT signaling pathway

## Abstract

This study aimed to assess the impact of conditioned medium from epidermal neural crest stem cells (EPI-NCSCs-CM) on functional recovery following spinal cord injury (SCI), while also exploring the involvement of the PI3K-AKT signaling pathway in regulating neuronal apoptosis. EPI-NCSCs were isolated from 10-day-old Sprague-Dawley rats and cultured for 48 h to obtain EPI-NCSC-CM. SHSY-5Y cells were subjected with H_2_O_2_ treatment to induce apoptosis. Cell viability and survival rates were evaluated using the CCK-8 assay and calcein-AM/PI staining. SCI contusion model was established in adult Sprague-Dawley rats to assess functional recovery, utilizing the Basso, Beattie and Bresnahan (BBB) scoring system, inclined test, and footprint observation. Neurological restoration after SCI was analyzed through electrophysiological recordings. Histological analysis included hematoxylin and eosin (H&E) staining and Nissl staining to evaluate tissue organization. Apoptosis and oxidative stress levels were assessed using TUNEL staining and ROS detection methods. Additionally, western blotting was performed to examine the expression of apoptotic markers and proteins related to the PI3K/AKT signaling pathway. EPI-NCSC-CM significantly facilitated functional and histological recovery in SCI rats by inhibiting neuronal apoptosis through modulation of the PI3K/AKT pathway. Administration of EPI-NCSCs-CM alleviated H2O2-induced neurotoxicity in SHSY-5Y cells in vitro. The use of LY294002, a PI3K inhibitor, underscored the crucial role of the PI3K/AKT signaling pathway in regulating neuronal apoptosis. This study contributes to the ongoing exploration of molecular pathways involved in spinal cord injury (SCI) repair, focusing on the therapeutic potential of EPI-NCSC-CM. The research findings indicate that EPI-NCSC-CM exerts a neuroprotective effect by suppressing neuronal apoptosis through activation of the PI3K/AKT pathway in SCI rats. These results highlight the promising role of EPI-NCSC-CM as a potential treatment strategy for SCI, emphasizing the significance of the PI3K/AKT pathway in mediating its beneficial effects.

**Graphical Abstract:**

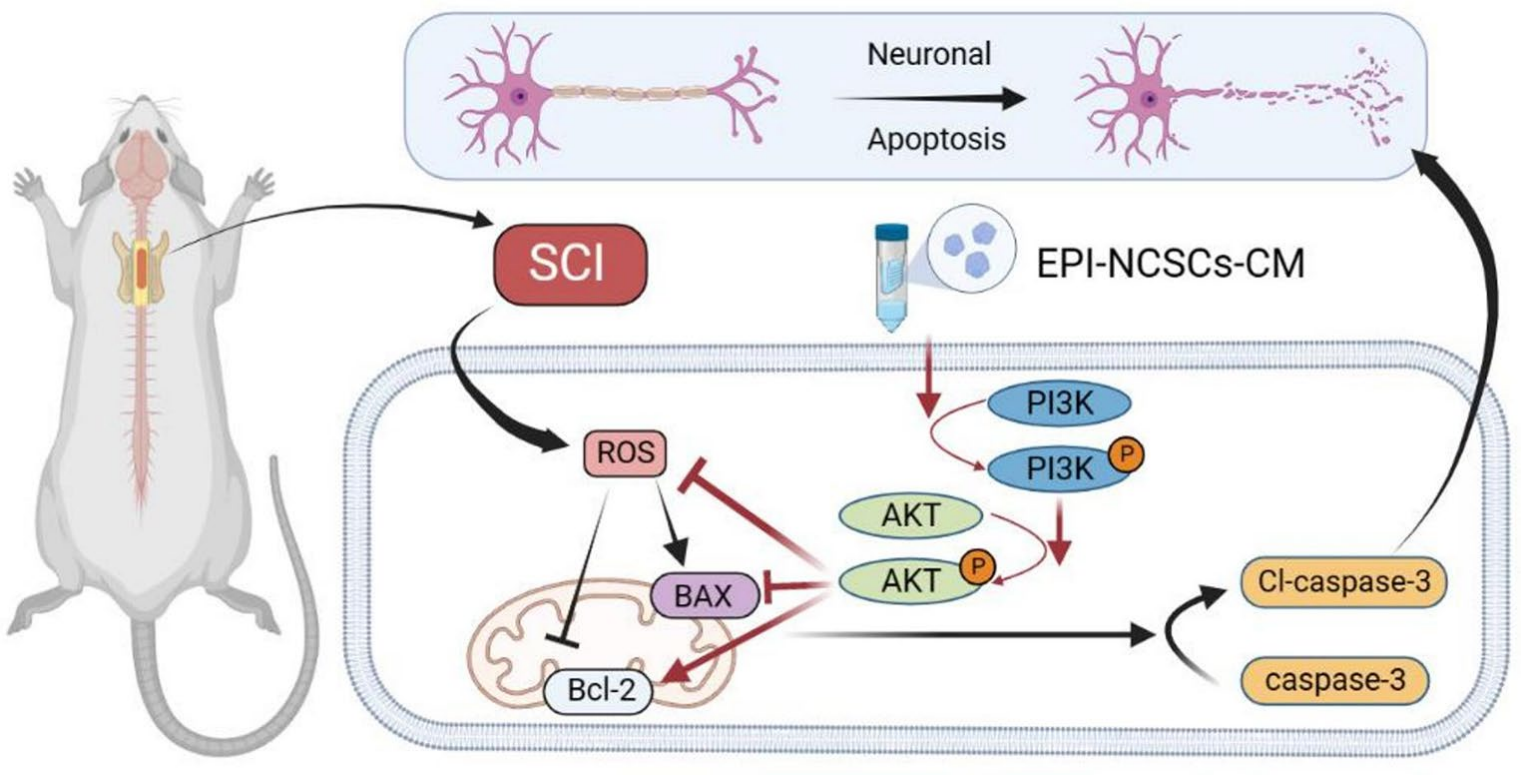

**Supplementary Information:**

The online version contains supplementary material available at 10.1007/s11064-024-04207-8.

## Introduction

Spinal cord injury (SCI) is a traumatic condition affecting approximately 300,000 people worldwide each year [1], often resulting in high disability and mortality rates due to irreversible spinal cord neuronal loss and axonal destruction [2]. Despite advances in treatments, such as surgery, medicines including riluzole [3, 4] and minocycline [5], hyperbaric oxygenation therapy, cell therapy, and rehabilitation strategies [6], effective repair options are lacking [7]. Therefore, there is an urgent need for further exploration of the SCI pathological mechanisms and repair strategies. SCI can be divided into primary and secondary injuries according to its pathophysiology. Primary injury is irreversible, while secondary injury involves a dynamic regulatory process, with apoptosis being a significant event. Kerr et al. first reported apoptosis in 1972 [8]. Neurons are vital spinal cord tissue components, and their loss directly leads to dysfunction after SCI. Previous studies have indicated neuronal apoptosis involvement in SCI pathophysiology and suggested its inhibition as a promising therapeutic strategy [9]. Excessive accumulation of reactive oxygen species (ROS) after SCI induces oxidative stress and neuronal apoptosis [10, 11]. Thus, inhibiting neuronal apoptosis is crucial for functional recovery after SCI.

Stem cell (SC) transplantation, including mesenchymal stem cells (MSCs), embryonic stem cells, and neural crest stem cells (NCSCs), can enhance functional recovery after SCI by promoting axonal growth and remyelination and balancing the microenvironment [12–14]. However, this therapy has drawbacks, such as poor survival after transplantation and severe side effects, including tissue immune rejection, teratogenicity, and tumorigenicity [15]. Research has shifted towards exploring the secretomes of SCs, including soluble bioactive molecules and vesicles, which regulate cellular processes [16, 17] due to their intense paracrine activity. SCs are regarded as “medicinal cell factories,” secreting various molecules with trophic and immunomodulatory effects [18]. These secreted factors can be found in the cell culture medium, known as conditioned medium (CM). The drawbacks of SC therapies have prompted investigations into SC-CM rather than SC transplantation for SCI’s repair. CM offers advantages, such as easy storage, longer shelf life, and fewer complications associated with cellular transplantation, making it a promising therapeutic candidate for treating SCI [19]. Studies have reported that CM administration promotes locomotor function recovery in rats [20–24]. For instance, our previous study demonstrated that CM derived from human dental pulp SCs reduces microglial pyroptosis by inhibiting the NLRP3/caspase-1/interleukin-1β pathway, thereby promoting neurological function recovery after SCI [24]. Wang et al. reported that bone marrow mesenchymal stem cell CM alleviates SCI by suppressing Gal-3 and NLRP3 expression [25]. In a systematic review and meta-analysis, Mahmoud et al. concluded that MSC-CM administration in SCI models improves motor recovery [26]. Fatemenh et al. investigated whether SC transplantation or SC-CM administration was more effective in treating SCI and found both to be equally effective after surveying existing publications [27]. Another clinical study using CM from MSCs in patients needing alveolar bone regeneration showed bone formation without systemic or local complications and no inflammatory cell infiltration [28].

Epidermal neural crest stem cells (EPI-NCSCs), first discovered by Sieber-Blum [29], have been identified as a promising source for SCI cell therapy [30]. These cells are extracted from the bulge area of hair follicles during adulthood, offering advantages such as easy accessibility and potential for autologous applications without immunological rejection. Moreover, their robust self-renewal and multipotency make them ideal for treating SCI [31]. Previous studies have indicated that the transplantation of EPI-NCSCs into injured rat spinal cords improves locomotor and sensory functions by releasing neurotrophic factors, angiogenic factors, and extracellular proteases, possibly through paracrine effects [32, 33]. In the latest research, Zhu et al. reported that exosomes derived from EPI-NCSCs combined with acellular nerve allografts could bridge facial nerve defects [34]. Afshin et al. combined human hair follicle-derived SCs and CM to treat a rat model of ischemic stroke, demonstrating that their combination therapy was more effective in reducing infarction and elevating target gene expression, especially in the hippocampus, thus highlighting the potential of CM in ischemic stroke treatment [35]. However, whether the CM derived from EPI-NCSCs promotes functional recovery in SCI rat models remains unknown.

In this study, we investigated the effectiveness of EPI-NCSC-CM in treating SCI both in vivo and in vitro, along with its underlying therapeutic mechanisms. We assessed its neuroprotective and therapeutic effects in a rat contusion model of SCI. We investigated its anti-neuronal apoptosis properties through ROS assay, analysis of apoptosis-related proteins, and TUNEL staining. Additionally, we explored the role of the phosphatidylinositol 3-kinase/protein kinase B (PI3K/AKT) signaling pathway, a major activator of inflammation and cell death in SCI [36].

## Methods and Materials

### Animal

Adult female Sprague-Dawley rats (230 ± 10 g) were obtained from the Vital River Laboratory Animal Technology Co., Ltd. (Beijing, China) and housed in a controlled environment with a 12-h dark/light cycle, regulated temperature, and humidity, with *ad libitum* access to water and food. The Institutional Animal Care and Use Committee of the Capital Medical University (Beijing, China) approved this study (Supplemental Approval No. AEEI-2023-154) on June 19, 2023, following the guidelines of the Committee for the Purpose of Control and Supervision of Experimentation on Animals (CPCSEA).

### Culture and Characterization of EPI-NCSCs

EPI-NCSCs were extracted from individual hair follicles of the rats’ whisker pads. After thrice washing the skin with phosphate-buffered saline (PBS), we separated and microdissected the bulge area from each follicle. The isolated bulges were cultured in 200 µM collagen-coated plates with essential medium-α (α-MEM, Sigma-Aldrich; USA) containing 10% fetal bovine serum (FBS, Gibco; USA), 5% day-11 chick embryo extract (US Biological; USA), and 1% penicillin/streptomycin (P/S, Gibco; USA). Cultures were incubated at 37ºC with 5% CO_2_, with half of the medium replaced daily. After 7–9 days, SC migration from the bulge area was observed. The bulging area was then removed, and Accutase (Gibco, USA) was used to detach and pass the cells. The procedure for EPI-NCSCs has been described in detail in a previous publication [37]. In brief, the cells were cultured in 200 µM collagen-coated plates at a density of 1 × 10^4^ cells/well in 4-well plates to verify their immunoreactivity to nestin and SOX10 using immunocytochemistry.

### Collection of EPI-NCSCs-CM

CM was collected as described previously [19, 20]. EPI-NCSCs-CM was obtained by culturing EPI-NCSCs (3.5 × 10^5^ cells/cm^2^) in serum-free DMEM/F12 (Gibco, USA) medium for 48 h. Control-CM (Con-CM) was collected after 48 h without culturing EPI-NCSCs. Samples were centrifuged at 500 ×g for 10 min to remove cell debris, filtered through a 0.22 μm syringe filter, then concentrated at 6000 ×g using Amicon Ultra-15 (Millipore Corporation, Bedford, MA, USA, 3000 kDa interception molecular weight) and stored at -80 °C for future experiments.

### Cell Viability Assays

CCK-8 (NCM Biotech Co., Ltd., CHINA) examined cell viability. SH-SY5Y cells (1 × 10^4^ cells/well) were pre-treated with Con-CM and EPI-NCSC-CM for 24 h in 96-well plates. Then, the medium was replaced with 100 µL of drug-containing medium, with the control receiving solvent. To determine the optimal H_2_O_2_ concentration, we exposed cells to varying concentration (50, 100, 200, 250, 400, and 500 µM) for 12 h. CCK-8 solution (10% v/v) was then added, and cells were incubated at 37ºC with 5% CO_2_ for 2 h. Absorbance (optical density, OD) at 450 nm was measured using Enzyme Markers (Thermo Fisher Scientific, USA), yielding an IC50 value of 205 µM for H_2_0_2_.

### SH-SY5Y Cell Apoptosis Model and EPI-NCSCs-CM Administration

SH-SY5Y cells (density: 2 × 10^4^ cells/cm^2^) were cultured in DMEM/F12 medium containing 10% fetal bovine serum (FBS), 1% P/S at 37 °C with 5% CO_2_. Prior to exposure to 205 µM H_2_0_2_ for 24 h to induce apoptosis, cells were pre-treated with Con-CM and EPI-NCSCs-CM for 12 h. In the subsequent phase, cells were pre-treated with a PI3K inhibitor (LY294002, 10 µM, MedChemEpress, China) for 12 h, followed by a 12 h pre-treated with EPI-NCSCs-CM before exposure to 205 µM H_2_O_2_ for 24 h.

### Calcein-AM/PI Staining

The calcein-AM/PI assay kit enables fluorescence-based cell viability assessment by simultaneously detecting live and dead cells using two probes. The probes measure intracellular esterase activity and plasma membrane integrity, akin to the Live/Dead^®^ Viability/Cytotoxicity Assay Kit. The assay was performed in 96-well plates, following the manufacturer’s instructions (C2015S, Beyotime). Cells were seeded at a density of 1 × 10^4^ cells/well in a 96-well plate at 37ºC in a 5% CO_2_ incubator. The Live/Dead^®^ Viability/Cytotoxicity Assay Kit stock solution was diluted with PBS to obtain a final concentration of 2 µM Calcein-AM and 4 µM PI, with 200 µL added to each well. SH-SY5Y cells were incubated in the dark for 30 min at 37ºC. After removing the assay buffer, images were captured using a laser scanning confocal microscope (Nikon, Japan).

### ROS Detection

ROS levels were measured using a DCFH-DA reactive oxygen fluorescent probe (Beyotime). SH-SY5Y cells were seeded into 24-well plates at a density of 2 × 10^4^ cells/well and treated with H_2_O_2_ or EPI-NCSC-CM. A 10 µM dyeing working solution was configured according to the product specification. After cell treatment, the culture medium was discarded, and 1 mL of 10 µM dyeing working solution was added to each well, incubating at room temperature for 20 min. After two washes with preheated PBS, fluorescence intensity was measured at an excitation wavelength of 485 nm using a fluorescence microscope (Nikon, Japan).

### Western Blot

Western blotting detected protein expression levels of Bcl-2, BAX, Cl-caspase-3, p-PI3K, PI3K, p-AKT, and AKT in SH-SY5Y cells and spinal cord tissues (0.5 cm on each side of the lesion). Proteins were isolated from cells and spinal cord tissues, quantified using the bicinchoninic acid (BCA) assay, and analyzed 3 days post-surgery. Total protein was extracted and quantified using the BCA method. Sodium dodecyl sulfate-polyacrylamide gel electrophoresis was conducted using the One-Step PAGE Gel Fast Preparation Kit (Vazyme). Proteins were transferred to nitrocellulose membranes (Bio-Rad, Hercules, CA, USA), then blocked with 5% fat-free milk for 1 h. Membranes were incubated overnight at 4 °C with primary antibodies, followed by a 1-h incubation at 37 °C with secondary antibodies. Protein bands were visualized using an ECL detection system (Bio-Rad), and intensities were analyzed using Image J2X (National Institutes of Health, Bethesda, MD, USA). Details of the primary and secondary antibodies used are listed in Table 1.

**Table 1:** Antibody information

Antibody	Host	Dilution	Catalog NO	RRID	Supplier	Application
BAX	Rabbit	1:1000	ab182734	-	Abcam	WB
Bcl-2	Rabbit	1:1000	ab182858	AB_2715467	Abcam	WB/IF
SOX-10	Rabbit	1:500	ab155279	AB_2650603	Abcam	IF
Nestin	Mouse	1:200	RAT-401	AB_1645181	Bioscience	IF
GAPDH	Mouse	1:5000	60004-1-Ig	AB_2107436	Proteintech	WB
Tuj1	Mouse	1:1000	ab78078	AB_2256751	Abcam	IF
Cl-caspase-3	Rabbit	1:1000	#9661	AB_2341188	CST	WB/IF
PI3K	Rabbit	1:1000	#4292	AB_329869	CST	WB
p-PI3K	Rabbit	1:1000	#4228	AB_659940	CST	WB
AKT	Mouse	1:2000	#2920	AB_1147620	CST	WB
P-AKT	Rabbit	1:1000	#9271	AB_329825	CST	WB
β-actin	Mouse	1:4000	HX1827	-	Huaxingbio	WB
Goat pAb to Rb IgG	Goat	1:500	ab150077	AB_2630356	Abcam	IF
Anti-Mouse IgG(H + L)	Goat	1:500	SA00007-1	AB_2889940	Proteintech	IF
HRP-Goat anti-Mouse IgG	Goat	1:5000	GB23301	AB_2904020	Servicebio	WB
HRP-Goat anti-Rabbit IgG	Goat	1:5000	ZB-2301	AB_2747412	ZSGB-BIO	WB

### Establishment of SCI Contusion Model and Treatment

Rats (*n* = 120) were randomly divided into three groups: sham (Sham), SCI + Con-CM (SCI group), and SCI + EPI-NCSC-CM (EPI-NCSC-CM group) (*n* = 40). Laminectomy and weight loss leading to SCI were performed as previously described [38]. Briefly, rats undergoing surgery were anesthetized with isoflurane (Lunan Pharmaceutical Group Corporation, Linyi, China, 4% for induction, 2% for maintenance). A midline incision exposed the T10 lamina, which was excised using a bone masseur (FST; Dusseldorf, Germany) to expose the spinal cord. A weight-dropping experiment used an IMPACTOR MODEL III (State University of New Jersey, New Jersey, USA) with rod parameters of 25 mm height, 10 g weight, and 3 mm diameter. The incisions were sutured. Only laminectomy was performed on sham group rats. After spinal cord contusion, rats displayed bilaterally hind limb paralysis, confirming successful model creation. Manual bladder emptying was performed twice daily until micturition function was restored. EPI-NCSCs-CM or Con-CM was intraperitoneally injected at 2.5 µL/g daily for 7 days post-surgery, as per previous literature [39–41]. The rats were housed under a 12-h light/dark cycle, *ad libitum* access to food and water, and at 22–25 °C and 30% humidity.

### Evaluation of the Functional Recovery of rats with SCI

Basso, Beattie, and Bresnahan (BBB) scores ranged from 21 (normal) to 0 (paralysis), and the inclined plane test was used to evaluate locomotion recovery at 1, 7, 14, 21, and 35 d after SCI [42]. Hind paw strength was tested on an inclined plane. A flat plate was constructed, and the angle was adjusted every 5° from the horizontal position (0°). Each animal was placed on a plate with its head facing left. The angle was gradually increased, and the maximum angle at which it could stay for 5 s without falling was recorded. The mean angle was obtained after repeating the test thrice. To analyze the gait of the model rats, we encouraged the animals to walk straight through a narrow path covered with white paper after immersing their four feet in ink (fore: red, hind: blue), and their footprints were recorded.

### Electrophysiological Test

Motor system recovery was assessed 35 days post-SCI using motor-evoked potentials (MEPs), following established protocols [43]. The rats were anesthetized using isoflurane, and an electrophysiological detector (Iridi Technology, Zhuhai, China) was used for the operation. MEPs were recorded with electrodes in the Achilles tendon and above the anterior fontanelle. Recovery analysis included amplitude and peak latency.

### Immunofluorescence (IF) Staining

Spinal cord tissues were harvested at 3 and 35 days after SCI and fixed in paraformaldehyde for 12 h. After 72 h sucrose dehydration, 10-µm longitudinal sections were cut, including the injury site, for immunofluorescence and histology (CM1950, Leica, Weztlar, Germany). For immunofluorescence, sections were incubated overnight at 4 °C with primary antibodies, washed with PBS, and then incubated at 37℃ for 1 h with goat anti-rabbit IgG or goat anti-mouse IgG. 4′,6-Diamidino-2-phenylindole (Sakura, Torrance, Calif, USA) was applied for 30 s. Images were captured using a laser-scanning confocal microscope (Nikon, Tokyo, Japan). Fluorescence intensity was evaluated using the ImageJ software. Antibody details are listed in Table 1.

### Hematoxylin-eosin (H&E) Staining

Cavitation areas were assessed using laser scanning confocal microscopy and hematoxylin-eosin staining (H&E staining), following the manufacturer’s protocol (C0105, Beyotime, Chengdu, China). For H&E staining, sections were stained with hematoxylin for 2 min, differentiated for 10 s, rinsed with running water, and then immersed in eosin dye solution for 1 min. After dehydration with increasing ethanol concentrations (70%, 80%, 90%, 95%, 100%), sections were sealed with neutral gum and a cover glass. Tissue loss area to spinal cord area ratio (axial plane) and relative area loss (sagittal plane) were calculated.

### Nissl Staining

A Nissl Body Staining Kit (Servicebio) assessed viable neuron counts in injured cords 35 days post injury (dpi). Sections were stained with cresol violet for 10 min, dehydrated in alcohol (70%, 80%, 95%, and 100%), fixed with neutral balm, and visualized under a microscope (Nikon, Tokyo, Japan).

### TUNEL Staining

The TUNEL staining kit (Beyotime, Beijing, China) can detect neuronal apoptosis. Cells and spinal cord tissues (10-µm longitudinal sections, including the injury site) were incubated with terminal deoxynucleotidyl transferase (TDT, Beyotime), deoxyuridine triphosphates (dUTP, Beyotime), and buffer at 37 °C for 1 h, followed by DAPI staining, and visualization under a fluorescence microscope (Nikon, Tokyo, Japan).

### Statistical Analysis

GraphPad Prism 9.0 (GraphPad Software, Inc., San Diego, CA, USA) was used to analyze the data. At least three animals per group were included for in vivo experiments, with evaluators blinded to subgroups. One-way analysis of variance (ANOVA) with Tukey’s post hoc tests was used to compare multiple groups, while repeated-measures two-way ANOVA with Tukey’s post hoc test was used for functional assessments (BBB scores and inclined plane). Mean ± standard error represented numerical data, with a statistical significance at *p* < 0.05.

## Results

### Extraction and Characterization of EPI-NCSCs

**Fig. 1:**
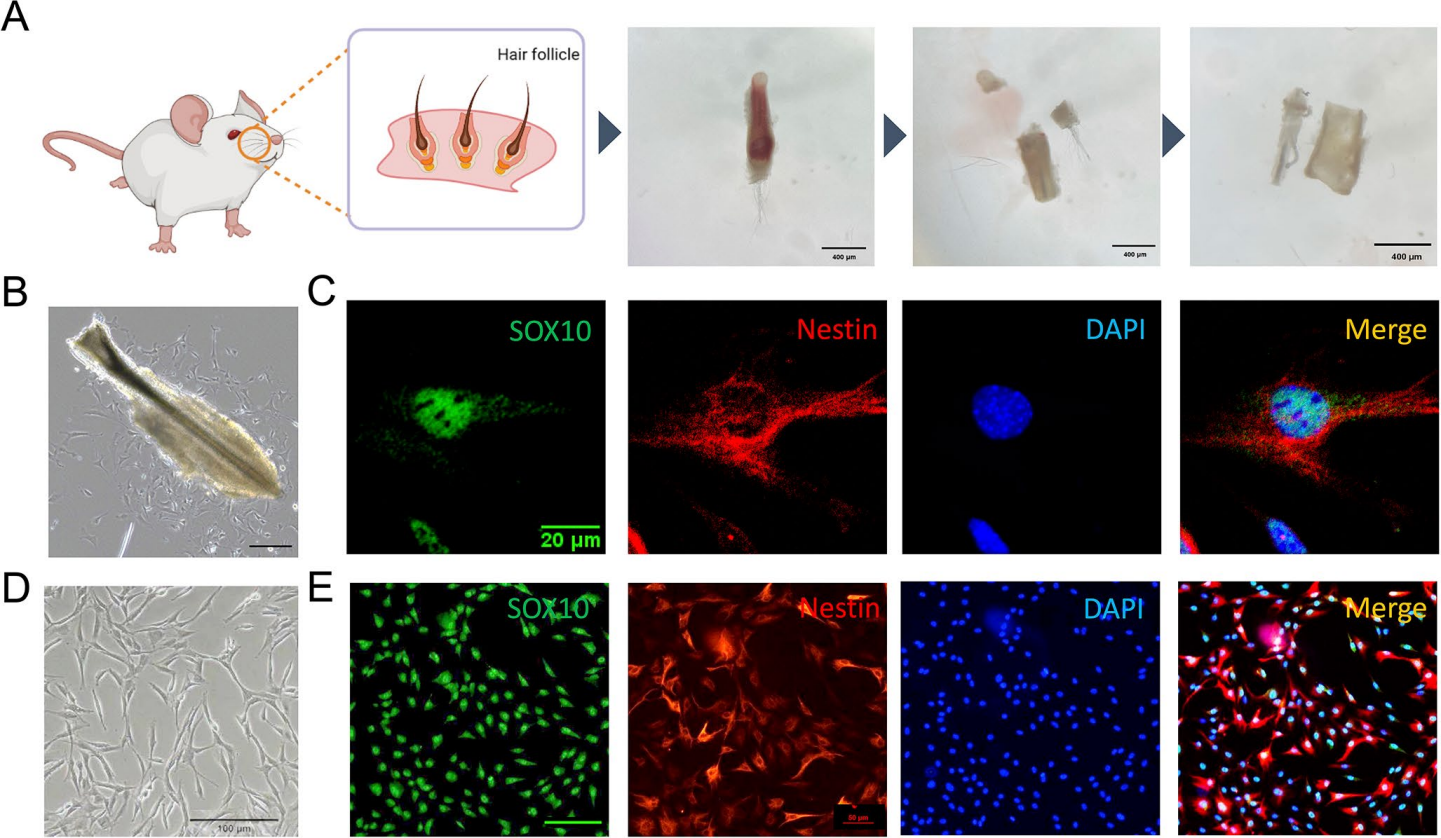
The isolation and characterization of EPI-NCSCs from rats. (**A**) The schematic diagrams of the microdissection of hair follicle explants from rat whisker pads. Briefly, the unilateral whisker pad was isolated from the rat. Afterward, single hair follicle was extracted. The top and bottom parts were removed to release the blood within the sheath. The connective tissue capsule surrounding the hair follicle explant was removed and the hair follicle explant was obtained. Scale bar: 400 μm. (**B**) Bright field image showing that most EPI-NCSCs emigrated from the hair follicle explant on day 4. Scale bar: 100 μm. (**C**) Immunofluorescence analysis of primary EPI-NCSCs (*n* = 4). Scale bar: 20 μm. (**D**) Bright field image showing the similar morphology of EPI-NCSCs at passage 3. Scale bar: 100 μm. (**E**) Immunofluorescent photographs showing that the EPI-NCSCs at passage 3 were positive for NCSC markers Nestin and Sox10 (*n* = 4). Scale bar: 100 μm

To extract and characterize EPI-NCSCs, we isolated rat whisker pad follicles and washed them with PBS thrice. The bulge area was obtained via microdissection (Fig. 1A). with most EPI-NCSCs migrating from the hair follicle explants after 7–9 days of culture (Fig. 1B). Immunofluorescence staining confirmed EPI-NCSC labelling via Nestin and Sox10 markers (Fig. 1C). Passaged EPI-NCSCs maintained morphology and immunoreactivity similar to primary cells (Fig. 1D, E). The ratio of SOX10 + Nestin co-expressing cells was stable during expansion (primary EPI-NCSCs: *n* = 4, mean co-expressing cell rate: 95.56%; passage 3 EPI-NCSCs: *n* = 4, mean co-expressing cell rate: 90.76%).

### EPI-NCSCs-CM Promotes the Functional Recovery after SCI

**Fig. 2:**
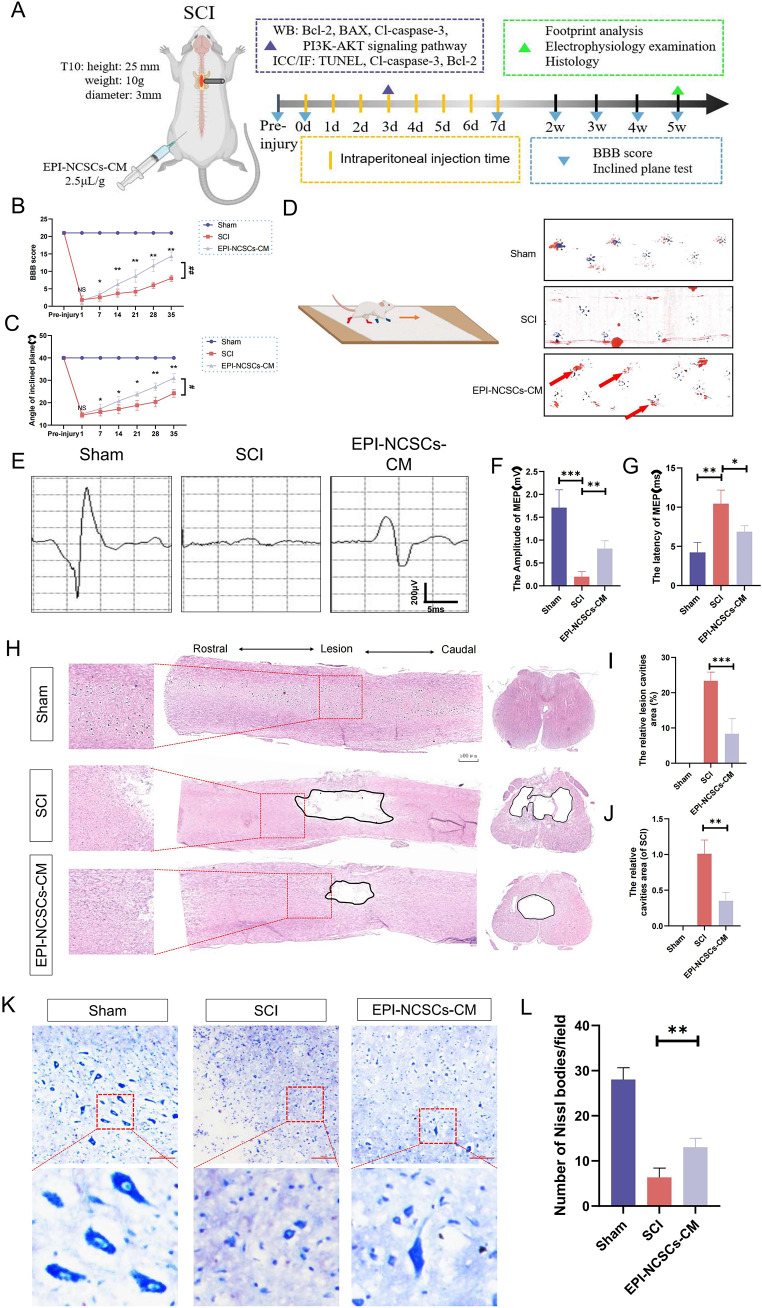
EPI-NCSCs-CM promotes the functional and histological recovery after SCI in rats. (**A**) The experimental design of the in vivo study. (**B**) BBB scores on day 1, 7, 14, 28 and 35 after SCI (*n* = 6). (**C**) Inclined plate test on days 1, 3, 7, 14, 21, 28 and 35 after SCI (*n* = 6). (**D**) Footprint analysis was performed to assess hindlimb motor function recovery. Forelimb footprints are shown in blue and hindlimb footprints are shown in red (*n* = 4). (**E**) The electrophysiology of each group was detected (*n* = 6). (**F**, **G**) Quantitative analysis of the amplitude and latency of motor evoked potential in different groups. (**H**-**J**) Images and quantification of H&E-stained cavity areas in longitudinal and transverse sections at 35 days post injury (*n* = 3). Scale bar: 500 μm. (**K**) Nissl staining of a transverse section of the spinal cord in different groups (*n* = 3). The box area of the higher magnification showing the healthy large-diameter Nissl-positive neurons. Scale bar: 100 μm. (**L**) The numbers of surviving neurons in the spinal cord tissue in each group were evaluated at 35 days post injury. Data expressed as Mean ± SD, ***p < 0.001, **p < 0.01, *p < 0.05 vs. SCI group. SCI vs. EPI-NCSCs-CM: ^##^*p* < 0.01, ^#^*p* < 0.05

The in vivo experimental design is shown in Fig. 2A. BBB scores, inclined plane test, electrophysiological recordings, and footprint analysis evaluated rat locomotor recovery over 35 days post-surgery. BBB scores decreased rapidly and persistently in the SCI group compared to the sham group, indicating significant motor impairment. EPI-NCSC-CM administration significantly increased BBB scores from day 7 post-SCI compared to Con-CM treatment. This beneficial effect persisted throughout the experiment (Fig. 2B). Moreover, EPI-NCSC-CM significantly increased the highest inclination angle in the inclined plane test (Fig. 2C), suggesting improved motor recovery. After 35 days, footprint analysis showed clear footprints in sham rats, while SCI rats showed two obvious drag marks (red ink) on hind limbs. Rats treated with the EPI-NCSCs-CM group showed some coordinated movement, evidenced by several red footprints (red arrows; Fig. 2D). Five weeks after SCI, evoked potential conductivity was measured. MEP latency and amplitude significantly differed among the sham, SCI, and EPI-NCSC-CM groups (Fig. 2E). Specifically, compared to the SCI group, EPI-NCSCs-CM treated rats showed larger peak amplitudes (Fig. 2F) and shorter latency of the first positive deflection (peak) (Fig. 2G). These results indicate that EPI-NCSC-CM treatment improved compromised evoked potential conductivity post-SCI.

### EPI-NCSCs-CM Decreases the Cavity Area and Neuronal Loss after SCI

To further investigate EPI-NCSC-CM’s protective effect post-SCI, histological analyses of spinal cord tissues were performed 35 days post-SCI. Longitudinal and transverse sections, stained with H&E, were used to evaluate lesion volume. EPI-NCSC-CM significantly reduced the cavity area compared to the SCI group (Fig. 2H-J). Nissl staining visualized neuronal loss, revealing pyknosis with a collapsed Nissl body in the SCI group, contrasting with preserved neurons in the EPI-NCSC-CM group (Fig. 2K). The remaining neurosomes’ maximum diameter was significantly larger with EPI-NCSC-CM treatment compared to SCI alone (Fig. 2L). These results suggest EPI-NCSC-CM may reduce injured areas and neuronal loss post-SCI.

### EPI-NCSCs-CM Inhibits the Neuronal Apoptosis via PI3K-AKT Signaling Pathway

**Fig. 3:**
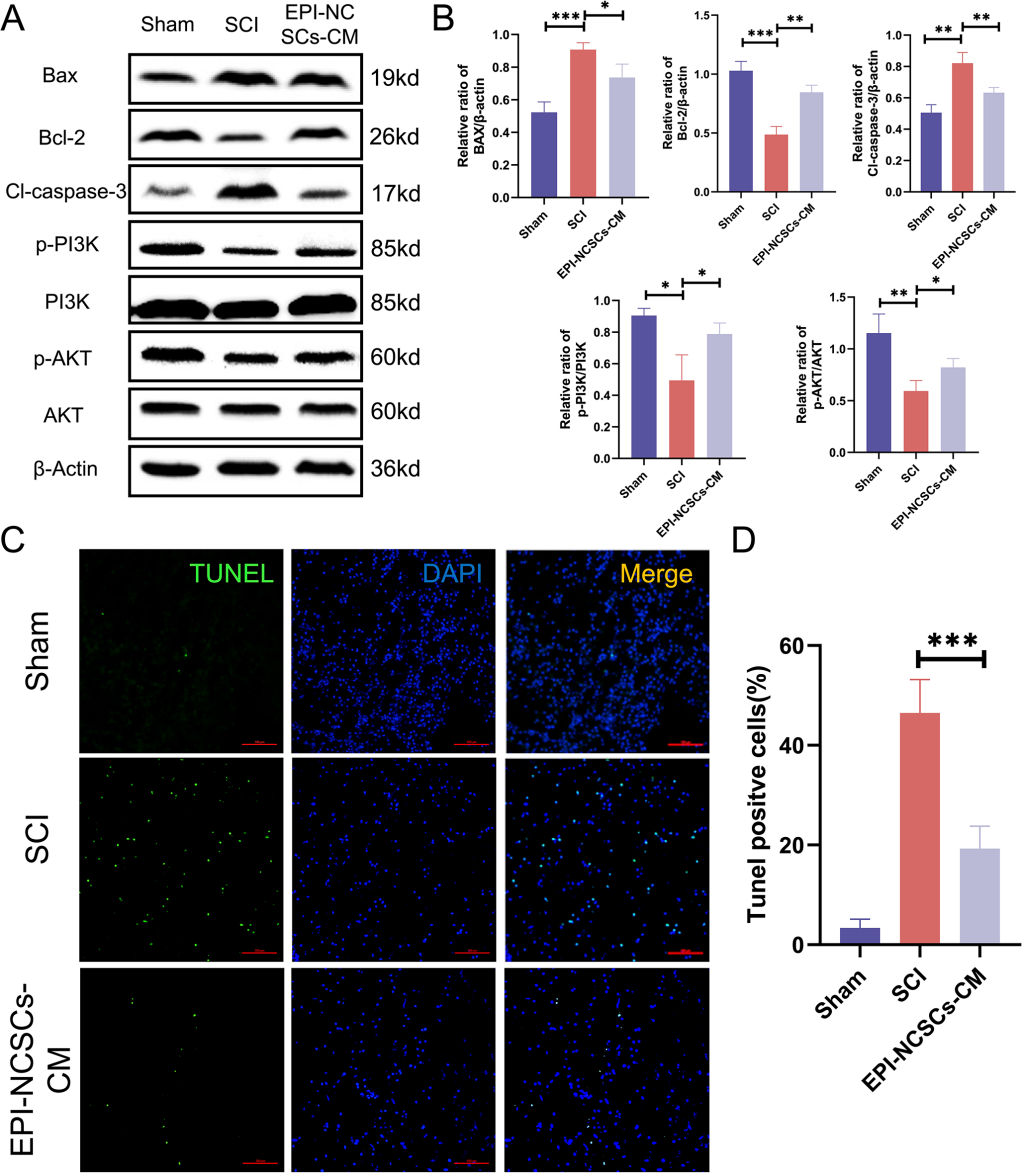
EPI-NCSCs-CM inhibits apoptosis after SCI. (**A**) Western blots of apoptosis markers and PI3K-AKT signaling pathway-related markers in spinal cord tissue of different groups (*n* = 3). (**B**) Statistical analysis of the relative expressions of BAX, Bcl-2, Cl-caspase-3, p-PI3K, PI3K, p-AKT, AKT in spinal cord tissues. (**C**) TUNEL staining of the transverse section of the spinal cord tissue in each group (*n* = 3). Scale bar: 100 μm. (**D**) Statistical analysis of the TUNEL positive cells in different groups. Data are expressed as Mean ± SD, ***p < 0.001, **p < 0.01, *p < 0.05 vs. SCI group

SCI induces apoptosis in surrounding nerve cells. Our results showed decreased anti-apoptotic Bcl-2 protein and increased pro-apoptotic BAX and Cl-caspase-3 proteins in injured spinal cord tissues (Fig. 3A, B). EPI-NCSC-CM treatment restores Bcl-2 expression and downregulates BAX and Cl-caspase-3 (Fig. 3A, B). Furthermore, TUNEL staining showed increased TUNEL-positive apoptotic cells post-SCI, mitigated by EPI-NCSCs-CM treatment (Fig. 3C, D), indicating reduced SCI-induced apoptosis. To determine whether EPI-NCSC-CM anti-apoptotic effects involve the PI3K-AKT signaling pathway, we examined p-PI3K, PI3K, p-AKT, and AKT protein levels 3 days post-SCI using western blotting. Results showed significantly downregulated p-PI3K/ PI3K and p-AKT/AKT proteins post-SCI, restored by EPI-NCSC-CM treatment (Fig. 3A, B).

**Fig. 4:**
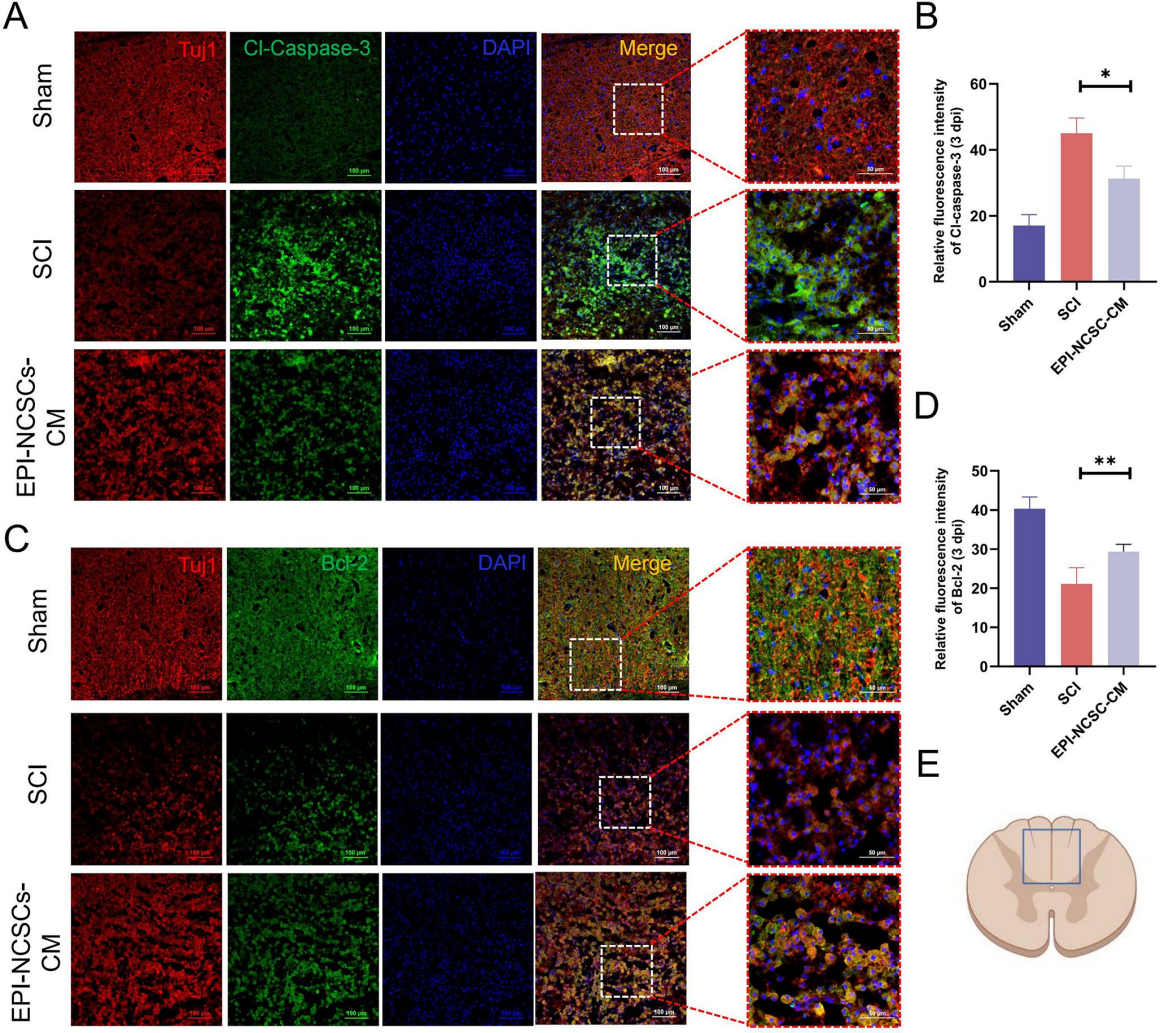
EPI-NCSCs-CM attenuates neuronal apoptosis after SCI. (**A**) Double immunofluorescence staining labeled Cl-Caspase-3 (green) and Tuj1 (red) on transverse sectioned tissue in each group at 3 days post-injury (*n* = 3). (**B**) Quantitative analysis of the fluorescence intensity of Cl-caspase-3 in neurons. (**C**) Double immunofluorescence staining labeled Bcl-2 (green) and Tuj1 (red) on transverse sectioned tissue in each group at 3 days post injury (*n* = 3). (**D**) Quantitative analysis of the fluorescence intensity of Bcl-2 in neurons. (**E**) Major areas of neuronal apoptosis were observed. Scale bar: 100 μm. Data are expressed as Mean ± SD, ***p < 0.001, **p < 0.01, *p < 0.05 vs. SCI group

Co-immunofluorescence staining of Tuj1 with Cl-caspase-3 and Bcl-2 examined EPI-NCSC-CM’s impact on neuronal apoptosis. The fluorescence intensity of Cl-caspase-3 in Tuj1 + cells significantly decreased in the EPI-NCSC-CM group compared to the SCI group (Fig. 4A, B). Conversely, Bcl-2 intensity in Tuj1 + cells significantly increased in the EPI-NCSC-CM group compared to the SCI group 3 days post-SCI (Fig. 4C, D). These findings suggest that EPI-NCSC-CM inhibits neuronal apoptosis via the PI3K-AKT signaling pathway after SCI.

### EPI-NCSCs-CM Protects SH-SY5Y Cells from H_2_O_2_-induced Cell Death

**Fig. 5:**
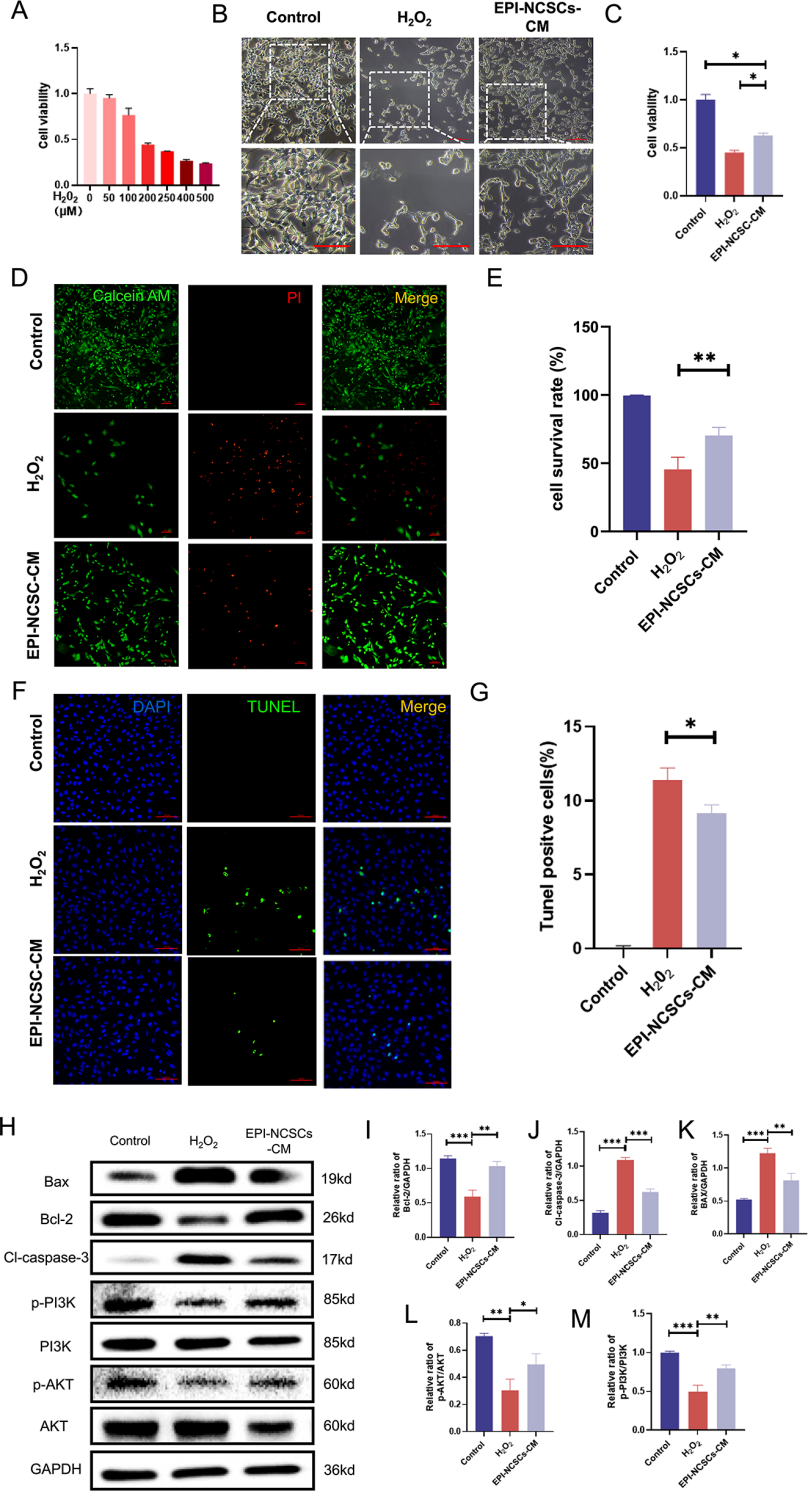
EPI-NCSCs-CM attenuates H_2_O_2_-induced apoptosis via regulating PI3K-AKT signaling pathway in SH-SY5Y cells. (**A**) Effects of different concentrations (0, 50, 100, 200, 250, 400, 500µM) of H_2_O_2_ on the viability of SH-SY5Y cells (*n* = 4). (**B**) Bright field images showing the morphology of SHSY-5Y cells in different groups; Scale bar: 100 μm (**C**) Effects of H_2_O_2_ and EPI-NCSCs-CM on the viability of SH-SY5Y cells (*n* = 4). (**D**) Calcein AM/PI staining of live cells (green) and dead cells (red) of Control, H_2_O_2_ and EPI-NCSCs-CM groups (*n* = 3). Scale bar: 100 μm. (**E**) Quantitative analysis of cell survival rate by Calcein AM/PI staining. (**F**) TUNEL staining of SH-SY5Y cells in different groups (*n* = 3). Scale bar: 100 μm. (**G**) Statistical analysis of TUNEL positive cells after treated by H_2_O_2_ and EPI-NCSCs-CM. (**H**) Western blot assays showing the expression level of the apoptosis related markers Bax, Bcl-2, Cl-caspase-3 and p-PI3K, PI3K, p-AKT, AKT protein levels in SH-SY5Y cells in different groups (*n* = 3). (I-M) Statistical analysis of the protein level in different groups. Data expressed as Mean ± SD, ***p < 0.001, **p < 0.01, *p < 0.05 vs. H_2_O_2_ group

To verify EPI-NCSC-CM’S on anti-neuronal apoptosis effect after SCI in vitro, we used a cellular model involving H_2_O_2_ in SH-SY5Y cells. We determined the optimized H_2_O_2_ concentration using a CCK-8 kit, testing 50–500 µM concentrations for 12 h. At 200 µM H_2_O_2_, cell viability decreased to 44.37 ± 1.72% (Fig. 5A), with an IC_50_ of 205 µM. We induced oxidative damage with 205 µM H_2_O_2_. Cells pre-treated with EPI-NCSCs-CM for 12 h showed morphological reversal of H2O2-induced damage, with increased viability (Fig. 5B, C). Calcein-AM/PI staining confirmed EPI-NCSC-CM significant inhibition of H_2_O_2_-induced cell death (Fig. 5D, E).

### EPI-NCSCs-CM Alleviates H_2_O_2_-induced Apoptosis in SH-SY5Y Cells

To further explore the protective mechanism, ROS assays, TUNEL staining, and western blotting confirmed EPI-NCSC-CM’s inhibitory effect on H_2_O_2_-induced apoptosis. DCFH-DA probe detected significant ROS increase post-H_2_O_2_ stimulation countered by EPI-NCSC-CM pre-treatment (Supplementary Fig. 1A). TUNEL staining revealed increased apoptosis post-H_2_O_2_, notably reduced by EPI-NCSC-CM (Fig. 5F, G). Western blot analysis showed H_2_O_2_-induced upregulation of BAX and Cl-caspase-3 and downregulation of the anti-apoptotic protein Bcl-2 was downregulated in SH-SY5Y cells, reversed by EPI-NCSC-CM addition (Fig. 5H-K). In summary, EPI-NCSC-CM effectively reduced H_2_O_2_-induced apoptosis in SH-SY5Y cells.

### PI3K/AKT Signaling Pathway Plays a role in the Inhibitory Effect of EPI-NCSCs-CM on H_2_O_2_-induced Apoptosis in SH-SY5Y Cells

To determine the involvement of the PI3K/AKT signaling pathway in H_2_O_2_-induced apoptosis and the protective effect of EPI-NCSC-CM, we analyzed key protein expression (p-PI3K, PI3K, p-AKT, and AKT) via western blot. Levels of p-PI3K and p-AKT significantly decreased in the H_2_O_2_ group compared to the controls. Conversely, their expression was higher in the EPI-NCSC-CM group compared to the H_2_O_2_ group (Fig. 5H, L, M).

**Fig. 6:**
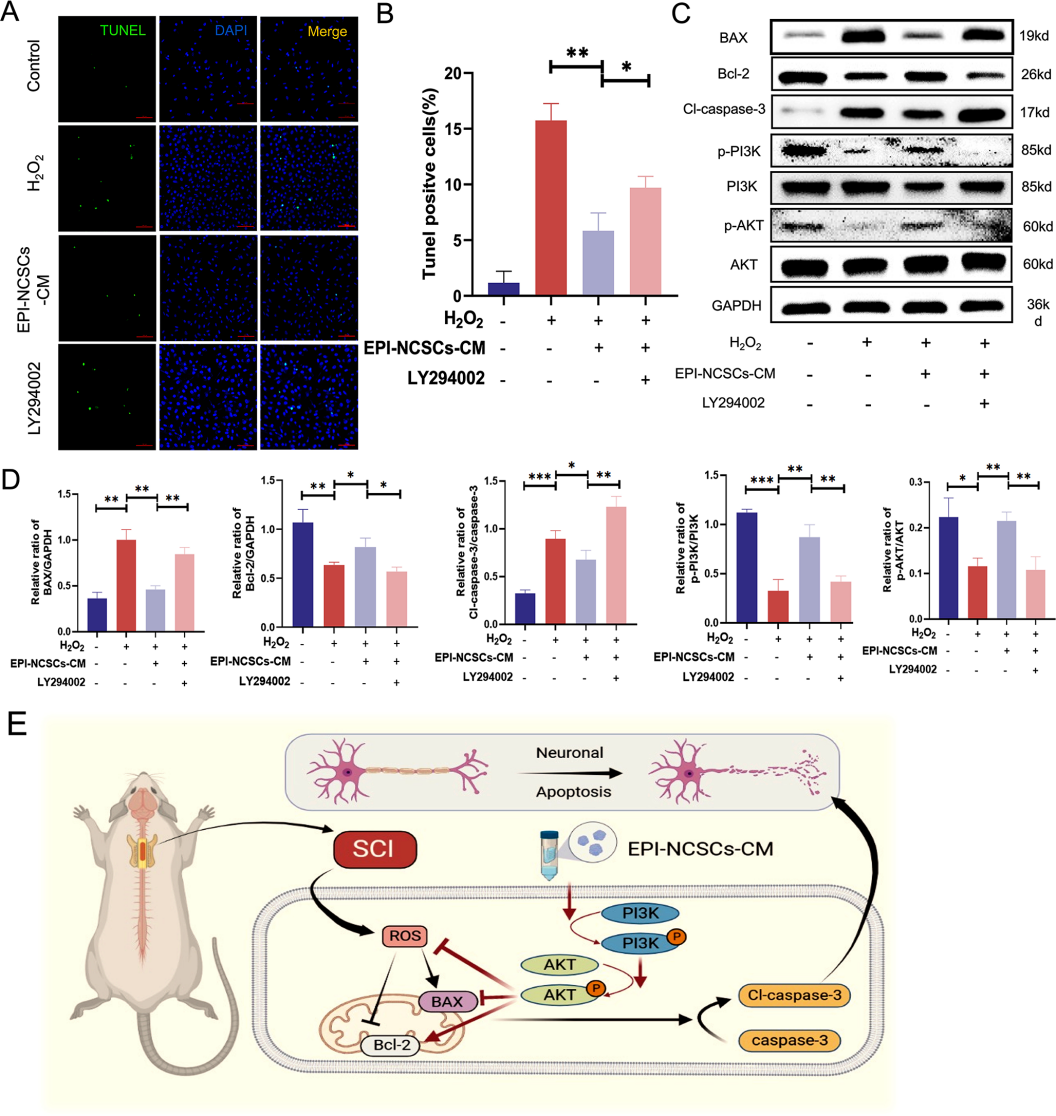
LY294002 reverses the inhibition of EPI-NCSCs-CM on apoptotic cell death of SHSY-5Y cells. (**A**) TUNEL staining of SH-SY5Y cells after treated by H_2_O_2_, EPI-NCSCs-CM and LY294002 (a PI3K inhibitor) (*n* = 3). Scale bar: 100 μm. (**B**) Statistical analysis of the TUNEL-positive cells in different groups. (**C**) Western blots of apoptosis markers and PI3K-AKT signaling pathway-related markers in SH-SY5Y cells (*n* = 3). (**D**) Statistical analysis of the relative expression of BAX, Bcl-2, Cl-caspase-3, p-PI3K, PI3K, p-AKT, AKT in SHSY-5Y cells. (**E**) The schematic illustrates the mechanism that EPI-NCSCs-CM regulates the neuronal apoptosis after SCI. EPI-NCSCs-CM activates the PI3K/AKT signaling pathway, inhibits the neuronal apoptosis, and ultimately promotes the functional recovery after SCI. Data are expressed as Mean ± SD. ***p < 0.001, **p < 0.01, *p < 0.05

To determine the role of the PI3K/AKT signaling pathway in EPI-NCSC-CM’s anti-apoptotic effect, we examined ROS level, pathway expression, apoptosis-related proteins, and apoptosis rate upon adding the PI3K inhibitor LY294002. ROS levels significantly increased post-inhibitor addition, partially attenuating the decrease observed in cells pre-treated with EPI-NCSC-CM (Supplementary Fig. 1C, D). TUNEL staining showed a higher proportion of late apoptotic cells in the LY294002-treated group compared to the EPI-NCSC-CM group and a decrease compared to the H_2_O_2_ group (Fig. 6A, B). These findings indicated that LY294002 partially counteracts the anti-apoptotic effect of EPI-NCSC-CM. Protein expression levels of BAX and Cl-caspase-3 decreased, while that of the anti-apoptotic protein Bcl-2 increased in H_2_O_2_-induced SH-SY5Y cells preconditioned with EPI-NCSC-CM. Furthermore, p-PI3K and p-AKT expression levels showed similar trends post-LY294002 treatment (Fig. 6C, D).

## Discussion

SCI causes profound physical, social, and vocational impacts on patients’ well-being [44, 45], with no cure, leading to irreversible loss of sensory and voluntary motor functions below the injury level. SCs offer promising therapeutic avenues, providing exogenous cell sources to compensate for the cell loss in SCI. However, cell transplantation has several challenges, including ethical issues, immunological responses, and low graft cell survival rates. Numerous studies suggest that transplanted cells can mitigate SCI by modulating the microenvironment through the secretion of various factors, offering a novel therapeutic approach [46]. Conditioned medium (CM) encompasses molecules secreted into the extracellular space, including soluble proteins, free nucleic acids, lipids, RNA, and extracellular vesicles such as apoptotic bodies, microvesicles, and exosomes. Numerous previous studies proved that CM alone derived from different stem cells, such as bone marrow mesenchymal stem cell (BMSC), human exfoliated deciduous teeth (SHED) and adipose-derived stem cell (ADSC) can enhance functional rehabilitation in SCI animal models [21, 47, 48]. Our own published research has also illustrated that CM from human dental pulp stem cells can effectively mitigate SCI by inhibiting microglial pyroptosis [24]. Hu et al. suggested that EPI-NCSCs release neurotrophic and angiogenic factors potentially contributing to SCI therapy [30]. Moreover, EPI-NCSCs exhibit significantly higher mRNA levels of BDNF, GDNF, and NGF than those in BMSCs [49]. Given the potentially heightened secretion capacity of EPI-NCSCs compared to other stem cell types, we hypothesized that EPI-NCSC-CM could be developed as an effective strategy for SCI treatment. To explore EPI-NCSCs’ paracrine effects, we utilized their secretomes for repairing SCI. Functional rehabilitation is crucial for assessing the therapeutic efficacy in SCI. Emerging strategies include immunotherapy [50], biological and engineering strategies for neural circuit reconstruction [51], and brain–spine interface utilization [52]. This study demonstrated that EPI-NCSC-CM promoted functional and histological recovery in SCI rats, aligning with Mahmoud et al.’s meta-analysis indicating that MSC-CM administration improved motor recovery in SCI models [26].

Extracellular vesicles (EV), including exosomes and microvesicles, have attracted considerable attention as promising tools for therapeutic applications, potentially surpassing CM. However, determining the preferred choice requires further investigation. Anna et al. applied quantitative proteomics to compare the protein composition of the CM and EV from adipose-derived stem/stromal cells and dermal fibroblasts, revealing distinct proteomes. As all analyses used equal protein amounts, factors more abundant or unique in EVs likely exist in CM in similar quantities per cell. Therefore, the authors concluded that CM may be more feasible owing to a complete product, simpler procedure, and reduced manipulation compared to EV production [53]. We found a similar viewpoint while extracting CM and EV. CM is easier to collect and store than EV. However, EV contains richer RNA components and proteins deserving further exploration. Furthermore, some studies aim to enhance the low concentrations of individual molecules and their therapeutic effects through external interventions. Suk et al. proposed a new approach using light to stimulate human adipose-derived SCs, thereby enhancing the production of angiogenic paracrine factors for angiogenesis [54].

SCI leads to irreversible dysfunction or loss of multiple cells and microenvironmental imbalances, with neuronal apoptosis being a crucial pathological mechanism in SCI [55, 56]. In this study, results from co-immunofluorescence and western blot showed that EPI-NCSC-CM protected spinal cord neurons from apoptosis. The expression levels of the apoptosis markers Cl-caspase-3, BAX, and Bcl-2 showed corresponding alterations after EPI-NCSC-CM administration in SCI rats. Furthermore, TUNEL staining suggested that EPI-NCSC-CM inhibited neuronal apoptosis following SCI. For the in vitro experiments, we used SH-SY5Y cells to establish a model of neuronal apoptosis induced by H_2_O_2_, commonly employed to mimic neuronal conditions [57–59]. Consistent with previous findings, excess cellular levels of ROS damaged proteins, nucleic acids, lipids, membranes, and organelles, leading to apoptosis. Our ROS assay showed that EPI-NCSC-CM inhibited neuronal apoptosis in vitro.

The PI3K/AKT signaling pathway is vital and closely related to the pathological process of SCI. Activation of this pathway can delay the inflammatory response, prevent glial scar formation, and promote neurological function recovery [60]. Numerous studies have reported the involvement of the PI3K/AKT signaling pathway in apoptosis in SCI [36, 61–63]. Therefore, we explored whether the PI3K-AKT signaling pathway participates in the therapeutic mechanism of EPI-NCSC-CM and found that expression of p-PI3K and p-AKT was downregulated after SCI in rats. However, EPI-NCSC-CM treatment upregulated their expression levels, indicating activation of the PI3K/AKT signaling pathway. To further confirm the upstream regulatory role of the PI3K/AKT signaling pathway, we used the PI3K inhibitor LY294002 to assess its role in the molecular mechanism of EPI-NCSC-CM in neuronal apoptosis. LY294002 administration reversed the inhibitory effect of EPI-NCSC-CM on apoptotic cell death in SH-SY5Y cells. This implies that EPI-NCSC-CM attenuated neuronal apoptosis by activating the PI3K/AKT signaling pathway. Besides, considering the pivotal role of the PI3K/AKT pathway in SCI protection, targeted activation of PI3K/AKT locally at the injured site of the spinal cord might be able to modulate its neuroprotective potential while minimizing the possible systemic side effects [62]. The precise target of PI3K/AKT signaling pathway in the spinal cord tissue can be realized through local administration of the PI3K/AKT specific activators, including bioactive factors or small molecules [64]. The targeted intervention not only allows for the precise targeting of therapeutic effects of activation of PI3K/AKT signaling pathway to the injured spinal cord area, but also maximizes the concentration of the activators within the local microenvironment. Furthermore, the localized approach also mitigates systemic side effects by minimizing the exposure of non-target tissues to possible harmful compounds, which provides more safety [65].

Our study had some limitations. First, we used SH-SY5Y cells to mimic primary neurons in vitro. Although the human neuroblastoma cell line is widely used in neuroscience research as a neuronal cell model [58], primary neurons are the most authoritative cell models for central nervous system research. Hence, future studies should use primary neurons to explore underlying molecular mechanisms. Secondly, we mainly focused on the effects of EPI-NCSC-CM on neurons; however, its effects on other types of cells within the spinal cord, such as glial cells, remain unclear. Glial cells, including astrocytes and microglia, are important components of the spinal cord. Whether EPI-NCSC-CM also affects glial cell survival needs to be investigated. Finally, component analysis of EPI-NCSC-CM was absent in this study. Since EPI-NCSC-CM contains various bioactive factors, growth factors, and cytokines secreted by cells under specific culture conditions, the specific purified components extracted from CM represent a more targeted approach, focusing on isolating and characterizing individual bioactive factors responsible for apoptosis after SCI. The complex nature of CM may hinder the identification of specific bioactive molecules responsible for therapeutic effects. Therefore, purification allows for precise control over the composition and concentration of active molecules, especially enhancing therapeutic efficacy while minimizing variability and unwanted side effects which will be beneficial for clinical transformation. However, we also worry about the extracted specific effective components may lack the corresponding synergistic interactions as present in CM, potentially limiting their overall efficacy in modulating complex biological processes such as apoptosis in SCI. However, the comparison of the efficacy and feasibility in treating SCI between CM and purified components should be elucidated for clinical application.

In conclusion, this study provides evidence that EPI-NCSC-CM can promote functional recovery by inhibiting neuronal apoptosis via the PI3K/AKT signaling pathway (Fig. 6E). These findings emphasize the promising role of EPI-NCSCs-CM as a candidate for SCI treatment and underscore the importance of the PI3K/AKT signaling pathway in mediating its beneficial effects. Further research is warranted to fully elucidate the underlying mechanisms and translate these findings into clinical applications for patients with SCI.

### Electronic Supplementary Material

Below is the link to the electronic supplementary material.

Supplementary Material 1

Supplementary Material 2

## Data Availability

The data and materials that support the findings of this study are available from the corresponding author, Xueming Chen, upon reasonable request.
