# Supplementary material for: Epidermal Neural Crest Stem Cell Conditioned Medium Enhances Spinal Cord Injury Recovery via PI3K/AKT-Mediated Neuronal Apoptosis Suppression
Source: Neurochem Res. 2024 Jul 18;49(10):2854–70. doi: 10.1007/s11064-024-04207-8 (PMC11365850; doi:10.1007/s11064-024-04207-8)
Supplement: Supplementary file 2 — Supplementary Material 2 [file 11064_2024_4207_MOESM2_ESM.docx]

**Supplementary figures legends**

**Supplemental figure 1.** **ROS detection by fluorescence staining.** (A) Representative images of DCFH-DA staining on SHSY-5Y cells treated with H_2_O_2_ and EPI-NCSCs-CM. Scale bar: 100 μm (B) Statistical analysis of the mean fluorescent intensity of different groups. (C) Representative images of DCFH-DA staining on SHSY-5Y cells treated by H_2_O_2_, EPI-NCSCs-CM and LY294002. Scale bar: 100 μm (D) Statistical analysis of the mean fluorescent intensity of different groups. (n=3) Data are expressed as Mean ± SD. ***p < 0.001, **p < 0.01, *p < 0.05.
